# Impact of COVID-19 lockdown measures on a cohort of eating disorders patients

**DOI:** 10.1186/s40337-020-00340-1

**Published:** 2020-11-02

**Authors:** Paulo P. P. Machado, Ana Pinto-Bastos, Rita Ramos, Tânia F. Rodrigues, Elsa Louro, Sónia Gonçalves, Isabel Brandão, Ana Vaz

**Affiliations:** 1 Psychotherapy and Psychopathology Research Unit – Psychology Research Centre, University of Minho, School of Psychology, Braga, Portugal; 2 Faculty of Medicine, Psychiatry Department, University Hospital Center of São João, 4200-319 Porto, Portugal

**Keywords:** COVID-19, Lockdown, Eating disorders, Clinical impairment

## Abstract

**Background:**

Lockdown implemented to prevent the COVID-19 spread resulted in marked changes in the lifestyle. The objective of the current study was to assess the impact of lockdown measures on a cohort of eating disorder (ED) patients being followed as part of an ongoing naturalistic treatment study.

**Methods:**

Ninety-nine patients aged 18 or older, currently or previously, in treatment at a Portuguese specialized hospital unit were contacted by phone and invited to participate in the current survey. Fifty-nine agreed to be interviewed by phone, and 43 agreed to respond to a set of self-report measures of ED symptoms, emotion regulation difficulties, clinical impairment, negative urgency, and COVID-19 impact, during the week after the end of the lockdown period.

**Results:**

Data showed that of the 26 patients currently in treatment: 8 remained unchanged (31%), 7 deteriorated (27%), and 11 reliably improved (42%). Of the 17 participants not currently in treatment: 3 deteriorated (18%), 9 remained unchanged (53%), and 5 (29%) improved after the lockdown period. The Coronavirus Impact Scale showed that most patients considered their routines moderately or extremely impacted, experienced stress related to coronavirus, and showed difficulty in maintaining physical exercise and feeding routines. Results suggest that higher impact of COVID-19 lockdown was significantly correlated with eating disorder symptoms and associated psychopathology, impulsivity, difficulties in emotion regulation and clinical impairment measured at post-lockdown. In addition, the impact of COVID-19 and lockdown measures on clinical impairment was mediated by difficulties in emotion regulation.

**Conclusions:**

Findings suggest that some ED patients may experience worsening of their condition, especially if associated with difficulties in emotion regulation, and these difficulties might be exacerbated in the context of a stressful crisis and lockdown measures, highlighting the need for intervention strategies to mitigate its negative impact.

## Plain English summary

The current study assessed the impact of the COVID-19 pandemic crisis and the government implemented lockdown measures on a group of patients diagnosed with eating disorders, attending a specialized treatment center in Portugal. We found that those struggling with eating disorders may experience worsening of their condition, especially if they tend to have difficulties in emotion regulation, and their living routines and access to care are disrupted. This can be especially the case during stressful and disrupting events like when the state of emergency was declared in Portugal to curb the spread of COVID-19.

## Background

Lockdown measures implemented by the government to prevent the coronavirus disease 2019 (COVID-19) pandemic spread resulted in marked changes in the lifestyle of the general population (e.g., closure of schools, businesses, gyms, and restaurants). During the confinement period, people were instructed to stay at home, refrain from unnecessary outings, leaving home was allowed to shop for basic needs (e.g., food, and medication), short walks around home for light exercise or walk dogs.

People with eating disorders might be disproportionally affected both by the pandemic crisis and the mitigating measures implemented to contain it [1]. For example, data, from a pilot study in Spain [2], showed that 12 out of 32 patients reported impairments in their eating disorder (ED) symptomatology, and most presented worries about increased uncertainty in their lives, being it, work, treatment, or loved ones related. Another survey [3], carried in Australia, showed that in a group of participants with ED, there was an increase in restricting, binge eating, purging, and exercise behaviors.

Rodgers and colleagues [4] proposed three pathways by which the COVID-19 pandemic might cause ED symptomatic exacerbation: 1) disruptions of daily routines, and constrains in outdoors, and social activities, that are useful in terms of emotional regulation; 2) exposure to ED-specific and anxiety-provoking media content; and 3) emotional distress caused by fears of contagion and increased health concerns. The combination of these pathways to increased risk combined with a potential lack of availability of protective factors, like social support and access to care have the potential to affect in a unique way people with eating disorders. Social distancing, lack of in-person social contact, and confinement to own home may increase negative affect, that associated with difficulties in emotional regulation might pose specific challenges to people with eating disorders.

However, results regarding the impact of lockdown in eating disorders are not consistent. For instance, most recent preliminary research [5] showed that patients with a diagnosis of Anorexia Nervosa reported significant decreases on their eating symptoms, after confinement due to COVID-19. However, these results were based on retrospective self-reported evaluation of symptoms using a novel instrument.

There is currently an urgent need for research addressing the mental health impact of the COVID-19 crisis, especially amongst those already suffering from a mental disorder [6], and an assessment of potential symptomatic mitigating or aggravating factors. The objective of the current study was to assess the impact of COVID-19 and lockdown measures on a cohort of ED patients being followed as part of an ongoing naturalistic treatment study.

## Methods

### Participants

Participants were recruited from an ongoing naturalistic longitudinal study conducted by our research team, focusing on treatment monitoring and follow up of patients with a diagnosis of eating disorders. In the context of that study, 99 patients aged 18 or older, currently or previously, in treatment at a Portuguese specialized hospital unit were contacted by phone in order to invite them to participate in the current survey.

Four patients refused to participate in this additional survey, and 36 weren’t reachable (10 had changed their contact number, 7 had their mobile phone turned off, and 19 did not answer the call). Fifty-nine participants agreed to participate and were interviewed to obtain clinical information regarding the lockdown period. In addition, they were asked to complete a set of self-report questionnaires, of which were received 43 responses.

Of these 43 participants, 26 reported to be currently in treatment.

For the purpose of this study, we used data collected in the last available evaluation prior to COVID-19 lockdown period. Data collection regarding the impact of COVID-19 lockdown started when the end of the state of emergency, in Portugal, was announced (April 30th), and finished two weeks later (May 15th). During this data collection period, the country was under a “state of calamity” where the population had the civic duty of home confinement, in spite of the reopening of some commercial establishments and public services, and a gradual deconfinement.

### Measures

*Clinical interview*: participants were asked about clinical information regarding the COVID-19 lockdown period, namely weight change during lockdown, current medication, if they were still under ED treatment, date of the last ED appointment, and current diagnosis.

*Eating Disorder Examination-Questionnaire (EDE-Q)* [7, 8]: is a 28-item measure used to assess eating disorder psychopathology focusing on the last 28 days. It generates four subscales (restraint, eating concern, shape concern, and weight concern) and a global score.

*Clinical Impairment Assessment (CIA)* [9, 10]: is a 16-item self-report questionnaire used to evaluate psychosocial impairment secondary to eating disorders, in the personal, social and cognitive domains, over the last 28 days. A higher score represents more impairment.

*Impulsive Behavior Scale-Negative Urgency Subscale* (*UPPS-P)* [11]: this subscale is composed of 12-item and is used to assess impulsivity when accompanied by negative emotions. Higher scores represent greater negative urgency.

*Difficulties in Emotion Regulation Scale - Short Form (DERS-SF)* [12, 13]: is an 18-item self-report questionnaire used to assess difficulties in emotion regulation. It contains six subscales: 1) Difficulties Engaging in Goal-Directed Behavior, 2) Lack of Emotional Clarity, 3) Nonacceptance of Emotional Responses, 4) Limited Access to Emotion Regulation Strategies, 5) Lack of Emotional Awareness, and 6) Impulse Control Difficulties and also generate a total score. Higher scores represent greater emotion regulation difficulties.

*Coronavirus Impact Scale (CIS)* [14]: is an 11-item questionnaire that assesses the extent to which COVID-19 pandemic changed participant’s lives in the following areas: routines, family income/employment, food access, mental health care access, access to social support, experience of stress related to COVID-19 pandemic, stress/family discord, personal diagnosis of coronavirus, immediate family members diagnosed with coronavirus, and extended family members and/or close friends diagnosed with COVID-19. In the current study, we also asked participants to rate how COVID-19 pandemic changed their lives in terms of two additional areas: feeding and physical exercise. Items were answered on a 4-point Likert scale, varying from 0 (“None/No change”) to 3 (“Severe”). Total score was computed by the sum of the 13 items of this adapted scale.

### Procedure

In order to assess the impact of COVID-19 lockdown in this population, all participants were contacted by phone. Those who accepted to participate were interviewed by a psychologist specialized in EDs assessment and were asked to complete a set of online questionnaires, using Google Forms. Telephone interviews were conducted in a semi-structured format by 2 interviewers (APB, EL). Information regarding the aims of the study and the voluntary nature of participation were provided. This study was approved by the ethical committee of the hospital involved.

### Statistical analysis

Statistical analyses were conducted using the IBM SPSS Statistics software, version 26.0. For the characterization of the sample, descriptive statistics were used.

We first assessed the impact of the lockdown period by computing change scores (CS) between post and pre-lockdown on the CIA total scores. A CS was considered reliable (reliable change) if exceeded the standard error of the measure.

Pearson correlations were conducted to test the association between variables under study and COVID-19 lockdown impact, and to inform the candidate variables to include in the mediation model. In order to examine the indirect effects of difficulties in emotion regulation (DERS-SF total) on the relationship between impact of COVID-19 lockdown and clinical impairment (CIA total), a regression-based bootstrapping approach with 5000 bias-corrected resamples and 95% confidence intervals was conducted using the PROCESS macro (v3.5) for SPSS (2020). A simple mediation model was tested in which: impact of COVID-19 lockdown was included as the independent variable (X); CIA total was included as the dependent variable (Y); and DERS-SF total was introduced in the model as the mediator variable (M). The inference on mediation was based on the analysis of the indirect effects’ estimates, considered significant if the interval between the lower and the upper bound of the 95% bias-corrected bootstrap confidence intervals did not contain zero [15, 16].

## Results

### Participant’s clinical status

Forty-three ED patients with ages ranging from 18 to 55 years old (M = 27.60, SD = 8.45) completed all assessments in the post confinement period. Most were women (95.3%). Twenty had a diagnosis of anorexia nervosa, 14 of bulimia nervosa, 2 binge eating disorder, and 7 other specified feeding or eating disorder.

During the COVID-19 lockdown period, only 8 participants still went to work regularly, 6 were unemployed, 5 in layoff, 9 in prophylactic isolation, and the remaining 13 were in telework or involved in remote academic work (e.g., online classes).

Thirteen ED patients (31.0%) reported a weight increase due to COVID-19 lockdown, 8 (19.0%) decrease on weight, 4 (9.5%) do not know their current weight, and 17 (40.5%) report having maintained the weight during this period.

Participants’ mean body mass index (BMI) based on self-reported weight and height during COVID-19 lockdown period was 21.58 kg/m^2^ (SD = 6.85), significantly different from the BMI in the last assessment 20.53 kg/m^2^ (SD = 6.60) (t_40_ = − 2.26, *p* = .030).

Table 1 presents in detail participants’ clinical and demographical data.

**Table 1:** Clinical and demographical characteristics

	N	%	M	SD	Min-Max
Age			27.60	8.45	18–55
Sex					
Women	41	95.3			
Men	2	4.7			
Diagnosis					
Bulimia Nervosa	14	32.6			
Anorexia Nervosa	20	46.5			
BED	2	4.7			
OSFED	7	16.3			
Currently on ED treatment					
No	17	39.5			
Yes	26	60.5			
Social Lockdown					
Yes, I’m in prophylactic isolation	9	20.9			
Yes, I’m working from home	3	7.0			
(telework)	5	11.6			
Yes, I’m in lay off	6	14.0			
Yes, I’m unemployed	10	23.3			
Yes, I’m having videoconferencing	8	18.6			
classes	0	0.0			
No, I still go to work regularly	2	4.7			
No, I still go to work sporadically					
Other					
Social Lockdown Period (weeks)			6.71	1. 64	2–9
Lockdown weight change					
Gained weight	13	31.0			
Lost weight	8	19.0			
Unchanged	17	40.5			
Doesn’t know or doesn’t care	4	9.5			

*Note. N* = 43, *BED* Binge Eating Disorder, *OSFED* Other Specified Feeding or Eating Disorder, *BMI* Body Mass Index, *ED* Eating Disorders, *M* Mean, *SD* Standard Deviation

### Impact of COVID-19 lockdown

Table 2 presents mean and standard deviation for all self-report measures in both assessment times, last available data for each participant and post-lockdown assessment. Group differences between pre-COVID-19, and post lockdown, were not significant after Bonferroni correction for multiple comparisons. Trying to extrapolate the possible impact of the lockdown period on our participants, at the individual level, we computed change scores on our measure of clinical impairment (CIA) between the previously available time point and the end of the lockdown period. Using Jacobson and Truax [17] criteria for reliable change, 16 patients improved since previously available assessment, 17 remained unchanged, and 10 deteriorated. Data showed that of the 26 patients currently in treatment: 8 remained unchanged (31%), 7 deteriorated (27%), and 11 reliably improved (42%). Of the 17 participants not currently in treatment: 3 deteriorated (18%), 9 remained unchanged (53%), and 5 (29%) improved after the lockdown period.

**Table 2:** Pre and post lockdown self-reported measures of eating disorder, clinical impairment, impulsivity, and difficulties in emotion regulation

	Previous Assessment M (SD)	COVID-19 Assessment M (SD)
EDE-Q Total	2.92 (1.57)	2.93 (1.58)
CIA Total	23.33 (12.94)	19.93 (13.16)
UPPS-P	2.59 (0.66)	2.65 (0.75)
DERS-SF Total	43.35 (15.94)	47.56 (14.85)

*Note. N* = 43, *EDE-Q* Eating Disorder Examination-Questionnaire, *UPPS-P* Impulsive Behavior Scale, *DERS-SF* Difficulties in Emotion Regulation Scale-Short Form, *CIA* Clinical Impairment Assessment

Results suggest that most participants, whether currently in treatment or not, considered that COVID-19 changed moderately to extremely their life in terms of routines, experiences of stress related to coronavirus pandemic, physical exercise and eating habits. The data are summarized in Table 3.

**Table 3:** Impact of COVID-19 lockdown in ED patients

	Currently in treatment				Total	
	Yes		No			
	N	%	N	%	N	%
Routines						
No change/Mild	6	23.1	4	23.5	10	23.3
Moderate/ Severe	20	76.9	13	76.5	33	76.7
Family Income/Employment						
No change/Mild	16	61.5	16	94.1	32	74.4
Moderate/ Severe	10	38.5	1	5.9	11	25.6
Food Access						
No change/Mild	26	100.0	16	94.1	42	97.7
Moderate/ Severe	0	0.0	1	5.9	1	2.3
Medical health care access						
No change/Mild	15	57.7	10	58.8	25	58.1
Moderate/ Severe	11	42.3	7	41.2	18	41.9
Mental health treatment access						
No change/Mild	14	53.8	13	76.5	27	62.8
Moderate/ Severe	12	46.2	4	23.5	16	37.2
Access to extended family and non-family social supports						
No change/Mild	21	80.8	15	88.2	36	83.7
Moderate/ Severe	5	19.2	2	11.8	7	16.3
Experiences of stress related to coronavirus pandemic						
None/Mild	11	42.3	7	41.2	18	41.9
Moderate/ Severe	15	57.7	10	58.8	25	58.1
Stress and discord in the family						
No change/Mild	21	80.8	15	88.2	36	83.7
Moderate/ Severe	5	19.2	2	11.8	7	16.3
Physical Exercise						
No change/Mild	10	38.5	8	47.1	18	41.9
Moderate/ Severe	16	61.5	9	52.9	25	58.1
Feeding						
No change/Mild	9	34.6	8	47.1	17	39.5
Moderate/ Severe	17	65.4	9	52.9	26	60.5

*Note.* N = 43

### Associations between the impact of COVID-19 lockdown, eating psychopathology and psychological distress during lockdown

Table 4 shows the correlations between impact of COVID-19 lockdown, eating psychopathology and psychological distress during lockdown. Results suggest that higher impact of COVID-19 lockdown was significantly correlated with eating disorder symptoms and associated psychopathology, impulsivity, difficulties in emotion regulation and clinical impairment measured at post-lockdown. Considering the significant correlations, we tested a model including impact of COVID-19 lockdown, clinical impairment and difficulties in emotion regulation.

**Table 4:** Correlations between impact of COVID-19 during lockdown, eating psychopathology and psychological distress variables

Variable		CIS	CIA	EDE-Q	UPPS-P	DERS-SF
1. CIS	Pearson’s r	–				
	Upper 95% CI	–				
	Lower 95% CI	–				
2. CIA Total	Pearson’s r	0.569***	–			
	Upper 95% CI	0.742	–			
	Lower 95% CI	0.323	–			
3. EDE-Q Total	Pearson’s r	0.451**	0.819***	–		
	Upper 95% CI	0.662	0.898	–		
	Lower 95% CI	0.175	0.687	–		
4. UPPS-P	Pearson’s r	0.380*	0.384*	0.392**	–	
	Upper 95% CI	0.611	0.614	0.620	–	
	Lower 95% CI	0.090	0.095	0.104	–	
5. DERS-SF Total	Pearson’s r	0.393**	0.626***	0.505***	0.682***	–
	Upper 95% CI	0.620	0.780	0.699	0.816	–
	Lower 95% CI	0.106	0.401	0.241	0.480	–

* *p* < .05, ** *p* < .01, *** *p* < .001

*CIS* Coronavirus Impact Scale, *CIA* Clinical Impairment Assessment, *EDE-Q* Eating Disorder Examination-Questionnaire, *UPPS-P* Impulsive Behavior Scale, *DERS-SF* Difficulties in Emotion Regulation Scale-Short Form

### The mediational role of emotion regulation difficulties in the relationship between the coronavirus impact and clinical impairment secondary to ED

**Fig. 1:**
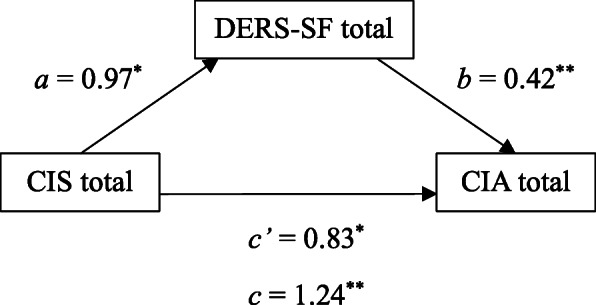
Statistical diagram of the simple mediation model of the relationship between the coronavirus impact and clinical impairment, mediated by emotion regulation difficulties. CIS - Coronavirus Impact Scale; DERS-SF - Difficulties in Emotion Regulation Scale-Short Form; CIA - Clinical Impairment Assessment. * *p* < .05, ** *p* < .01

The total effect of the impact of COVID-19 lockdown (CIS total) on clinical impairment (CIA total) was significant, c = 1.24, t = 4.43, *p* < .001. Also, the CIS total was positively associated to difficulties in emotion regulation (DERS-SF total), a = 0.97, CI [.26, 1.69], which was, in turn, positively associated with CIA total, b = 0.42, CI [.21, .64]. After introducing the mediator in the model, the association between the CIS total on CIA total remained significant, c´ = 0.83, t = 3.18, *p* = .003. Finally, the indirect effect of CIS total on CIA total via DERS-SF total was positive and statistically significant, ab = 0.41, CI [.11, .81]. The final model (Fig. 1), accounted for a total of 51% of the final variance of CIA total (Table 5).

**Table 5:** Model coefficients for the mediation model

	Consequent							
		*M* (DERS-SF total)				*Y* (CIA total)		
Antecedent		Coeff.	*SE*	*p*		Coeff.	*SE*	*p*
X (CIS total)	*a*	0.97	0.35	.009	*c’*	0.83	0.26	.003
*M* (DERS-SF total)		–	–	–	*b*	0.42	0.11	< .001
Constant	*i*_*M*_	35.07	5.02	< .001	*i*_*Y*_	−10.85	5.05	.038
		*R*^2^ = 0.15				*R*^2^ = 0.51		
		*F*(1, 41) = 7.51, *p* = .009				*F*(2, 40) = 21.20, *p* = < .001		

*Note.* N = 43, *CIS* Coronavirus Impact Scale, *DERS-SF* Difficulties in Emotion Regulation Scale - Short Form, *CIA* Clinical Impairment Assessment

## Discussion

To our knowledge, this was one of the first studies to examine the impact of COVID-19 pandemic and the associated confinement measures on a cohort of patients diagnosed with eating disorders. Because these patients were participating on a naturalistic study and there was previously available data on several aspects of their psychosocial functioning, it was possible to prospectively measure the potential impact of the COVID-19 pandemic and the lockdown confinement measures.

The COVID-19 pandemic and the lockdown-imposed measures had a significant impact on patients with eating disorders. The vast majority of patients reported that the lockdown measures had impacted, moderately to severely, their life routines. Around half of the patients reported to have significant difficulties assessing medical health care and even more so accessing mental health care. Other significant changes noted were related to exercise and eating routines. This change in life routines and the increased time spent at home, in a potentially triggering environment, might have created additional challenges to ED patients.

Curiously only a minority of patients reported moderate to severe difficulty in access to extended family and non-family social support. The fact that this was a relatively young cohort of patients with easy access to social media and internet-based communication tools, and the advertised benefit of keeping in touch with social support groups (i.e., social distancing does not mean social isolation) might have contributed to this fact.

Our data suggest that patients that experience significant changes in their living routines and report highest impact of COVID-19 crisis and lockdown measures, also experience increased psychological distress, which, in turn, may result in more disordered eating and clinical impairment. The changes imposed by the COVID-19 lockdown were significantly associated with clinical impairment. Most importantly, this impact was mediated by emotion regulation difficulties.

Although the COVID-19 crisis had impacted the general population as well, there are reasons to believe that those struggling with a mental health problem, and transient difficulties in accessing regular mental health care services, might be disproportionally affected by the pandemic crisis and associated measures [18]. However, further research that includes control group comparisons are needed to understand whether ED patients are significantly more affected than the general population.

To the best of our knowledge, this is one of the first studies to assess the impact of COVID-19 and lockdown measures in a cohort of ED patients that is not completely retrospective. The timing of the data collection, and the availability of data from pre-COVID-19 provided a unique opportunity to assess the impact of the pandemic crisis and associated lockdown measures. However, the current study has limitations, namely: 1) the use of a convenient, relatively small, sample based on an ongoing naturalistic study, 2) the use of phone interviews in the second wave of data collection as opposed to face-to-face in the first one; 3) the use of self-report measures and self-report height and weight that might have introduced some bias; 4) most patients that we approached, for various reasons, did not take part of the study; and, 5) findings reflect the experience of a sample of patients in Portugal.

## Conclusions

Those struggling with eating disorders may experience worsening of their condition, especially if associated with difficulties in emotion regulation, and these difficulties might be exacerbated in the context of a stressful crisis and lockdown measures. Given the need to plan for future adjustments to deal with COVID-19 is important to take these findings into consideration. Future interventions need to include self-help strategies that provide structure when usual routines are disrupted and adaptive emotion regulation strategies to deal with stressful events.

## Data Availability

The datasets used and/or analysed during the current study are available from the corresponding author on reasonable request.

## Abbreviations

ED: Eating Disorders
COVID-19: Coronavirus disease 2019;
EDE-Q: Eating Disorder Examination-Questionnaire
CIA: Clinical Impairment Assessment
UPPS-P: Impulsive Behavior Scale-Negative Urgency Subscale
DERS-SF: Difficulties in Emotion Regulation Scale - Short Form
CIS: Coronavirus Impact Scale
CS: Change scores
SPSS: Statistical package for the social sciences
